# Parametric investigation on mixing in a micromixer with two-layer crossing channels

**DOI:** 10.1186/s40064-016-2477-x

**Published:** 2016-06-21

**Authors:** Shakhawat Hossain, Kwang-Yong Kim

**Affiliations:** Department of Mechanical Engineering, Inha University, Incheon, 402-751 Republic of Korea

**Keywords:** Chaotic micromixer, Two-layer crossing channels, Mixing index, Navier–Stokes equations, Reynolds number

## Abstract

This work presents a parametric investigation on flow and mixing in a chaotic micromixer consisting of two-layer crossing channels proposed by Xia et al. (Lab Chip 5: 748–755, 2005). The flow and mixing performance were numerically analyzed using commercially available software ANSYS CFX-15.0, which solves the Navier–Stokes and mass conservation equations with a diffusion–convection model in a Reynolds number range from 0.2 to 40. A mixing index based on the variance of the mass fraction of the mixture was employed to evaluate the mixing performance of the micromixer. The flow structure in the channel was also investigated to identify the relationship with mixing performance. The mixing performance and pressure-drop were evaluated with two dimensionless geometric parameters, i.e., ratios of the sub-channel width to the main channel width and the channels depth to the main channel width. The results revealed that the mixing index at the exit of the micromixer increases with increase in the channel depth-to-width ratio, but decreases with increase in the sub-channel width to main channel width ratio. And, it was found that the mixing index could be increased up to 0.90 with variations of the geometric parameters at Re = 0.2, and the pressure drop was very sensitive to the geometric parameters.

## Background

The exponential demand for miniaturization in microfluidic applications highlights the significance of understanding the mechanism that controls mixing of fluid species at the microscale stage. The characteristic dimension of a microfluidic device is usually in a range from ten to several hundred micrometer, where the flow becomes laminar due to the low Reynolds number. Thus, the laminar behavior in the devices causes difficulty in mixing of fluids. Due to the low flow velocity, mixing primarily depends on the molecular diffusion between the fluids, which is very slow process. Diffusion mechanism is governed by the Fick’s law, where the mixing mass flux is proportional to the diffusion coefficient and concentration gradient. Microfluidic devises are widely applied for many chemical and biological practices, leading to the new ideas, such as bio-chip (Schwesinger et al. 1996), bio-MEMS (Linder 2001), microreactor (Hardt et al. 2005), and lab-on-a-chip (Erickson 2005). Rapid as well as efficient mixing is very important for almost all chemical and biological analyses. In order to fulfill the demand of efficient and rapid mixing in microchannels, a variety of passive and active micromixers have been developed so far (Nguyen and Wu 2005; Hessel et al. 2005). Active micromixers are coupled with external excitations by electroosmotic, dielectrophoresis, ultrasonic vibration, electrohydrodynamics, magnetic force, etc. (Hessel et al. 2005). Due to the additional parts for the excitations, the design and fabrication of the active micromixers are rather complex and expensive. However, passive micromixers do not use any exterior power source to induce disturbances. Thus, the passive micromixers can be easily incorporated in a composite microfluidic systems (Nguyen and Wu 2005; Hessel et al. 2005) due to the simplicity in their structures. In the lamination micromixer, mixing occurs by successive separation and rejoining of the fluid streams, which increase the interfacial area between the fluids (Gray et al. 1999). Recently, various techniques have been used to promote mixing in passive micromixers, for example, by introducing geometric adaptation in microchannels (Reyes et al. 2002; Manz et al. 1990; Weibel and Whitesides 2006).

Recently, computational fluid dynamics (CFD) has become very popular technique to analyze the mixing and fluid flow in micromixers. In recent years, many investigations have been performed to improve the mixing in a variety of passive micromixers; T-type micromixer (Gobby et al. 2001) and Y-type micromixer (Sahu et al. 2012), serpentine micromixers (Beebe et al. 2001; Hossain et al. 2009; Ansari and Kim 2009; Sahu et al. 2013), split-and-recombinination (SAR) micromixers (Lee and Lee 2008; Viktorov and Nimafar 2013; Nimafar et al. 2012a, b; Hossain and Kim 2014), patterned grooves micromixers (Ansari and Kim 2007; Hossain et al. 2010a, b), etc. It was exposed that shifting the inlet direction in a T-type micromixer did not appreciably improve the mixing performance (Gobby et al. 2001). In planner serpentine micromixer, sharp corner can produce center-rotating vortices at moderate Reynolds numbers (Re > 25), which promotes the agitating process to enhance the mixing performance (Beebe et al. 2001). Unluckily, at low Reynolds numbers, induced vortices in a micromixer decay sooner than they create an opportunity to considerably stir the mixing species.

A staggered herringbone grooves micromixers use a herringbone grooves pattern on the bottom of the channel, to induce lateral transports and thus to generate chaotic flows (Stroock et al. 2002a, b) to enhance the mixing performance. Hong et al. (2004) proposed a passive micromixer that employs the “Coanda effect” using two-dimensional Tesla structures to generate transverse dispersion. Tesla structure divides the fluid stream into the sub-streams, which recombine later. The splitting and recombining mechanism can create chaotic advection and improve the mixing, significantly. A parametric study of a modified Tesla structure was conducted by Hossain et al. (2010a, b) for a wide Reynolds number range from 0.05 to 40 with two geometric parameters. A staggered overlapping crisscross micromixer based on chaotic mixing principles was designed and fabricated by Wang and Yang (2006). Their numerical and experimental results show that the micromixer can generate chaotic flows to stretch and fold the fluid streams rapidly. Xia et al. (2005) proposed micromixers based on chaotic mixing principles, and performed numerical and experimental investigations of the micromixers. At very low Reynolds numbers, the proposed micromixers can manipulate the flow by splitting-and-recombining and stretching-and-folding which generate chaotic advection, and thus, significantly enhance the mixing. As the generation of chaotic advection does not depend on the inertial forces of fluids, the proposed micromixers worked well especially at low Reynolds number (Re = 0.2).

In the present work, a parametric study of the micromixer proposed by Xia et al. (2005) has been performed to systematically investigate the performance of the micromixer, which shows incredibly high mixing performance at very low Reynolds number. The sub-channel width, main channel width and channel depth were selected as the geometric parameters to be tested, and the mixing index was used as the performance parameter for mixing. The mixing index was evaluated using three-dimensional Navier–Stokes and mass conservation equations with a diffusion–convection model.

## Micromixer model

**Fig. 1:**
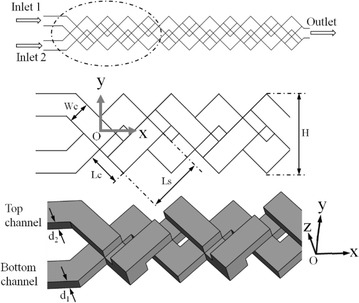
Schematic diagram of the micromixer with two-layer crossing channels and relevant parameters

A schematic diagram of the reference micromixer geometry proposed by Xia et al. (2005) is shown in Fig. 1. This micromixer consists of two layers of three-dimensional ‘‘X-shaped’’ segments. The diagonal channels have an angle of 45° with the inlets of the micromixer and both are perpendicular to each other. The channels are arranged in a periodic fashion. The sub-channel width is symbolized by Wc. For the parametric investigations, width of the main channel (H) was kept constant at 1.06 mm. The channels depth (D) can be expressed as d_1_ + d_2_, where d_1_ and d_2_ are the depths of the bottom and top channels, respectively. The reference values of Wc and D, are same as 0.3 mm, and the number of rhombic units is eleven. The symbols Lc and Ls indicate the lengths of connecting channels shown in Fig. 1.

In this work, a parametric study of the micromixer shown in Fig. 1 was performed to find the effects of two dimensionless geometric parameters, Wc/H and D/H on mixing performance. The design ranges and reference values of these parameters are depicted in Table 1. In the parametric study, if one parameter varies, the other parameter is fixed as the reference value.

**Table 1:** Geometric parameters and their ranges

Design variable	Lower limit	Upper limit	Reference value (Xia et al. 2005)
D/H	0.18	0.37	0.28
Wc/H	0.18	0.33	0.28

## Numerical formulation

To explore the flow structure along with mixing performance of the micromixer, the commercial CFD code ANSYS CFX-15.0 (CFX 15.0 2013) was employed, which solves Navier–Stokes equations through a finite volume approximation. The continuity and Navier–Stokes equations of fluid mixture solved in this work are represented as follows:

1 $$\nabla \cdot \vec{V} = 0$$

2 $$(\vec{V} \cdot \nabla )\vec{V} = \frac{1}{\rho }\nabla {\text{p + }}{\varvec{\upnu}}\nabla^{2} \vec{V}$$

where $\vec{V}$, ρ and **ν** correspond to the velocity, density, and kinematic viscosity of the fluid mixture, respectively. To examine the mixing mechanism, water at 25 °C and a water-dye solution were used as the working fluids. For each fluid component with constant viscosity and density, the mass transport equation of advection–diffusion type (Bird et al. 1960), is formulated as follows;

3 $$\vec{V} \cdot \nabla C_{i} = \alpha \nabla^{2} C_{i}$$

where α and *C*_*i*_ specify the diffusivity coefficient and concentration of the fluid component, respectively. For modeling of diffusive mixing, the scalar transport equation was used by many researchers for different micromixers (Chung et al. 2008; Cortes-Quiroz et al. 2010; Hinsmann et al. 2001; Afzal and Kim 2015).

To solve the above equations, the following boundary conditions were considered. Pure water at 25 °C was introduced at the Inlet 1 and the water-dye solution enters at Inlet 2. Constant velocity was specified at the inlets, while zero static pressure was assigned at the outlet. No-slip condition was employed at the walls. Physical properties of the water used in this work were same as those in the previous work (Xia et al. 2005). Diffusivity coefficient of the water-dye mixture was 1 × 10^−11^ m^2^/s. And, the density (*ρ*) and dynamic viscosity (*µ*) of water were 997 kg/m^3^ and 8.8 × 10–3 kg/m-s, respectively (Kirby 2010).

To conduct the numerical analysis, an unstructured tetrahedral grid system was created using ANSYS ICEM 15.0. The numerical diffusion error is generally induced due to discretization of convection terms in the Navier–Stokes equations. Higher-order upwind schemes can be used to minimize the numerical diffusion (Hardt and Schöndfeld 2003). To discretize the advection terms of the governing equations, a high-resolution scheme of second-order approximation was utilized in this work. By the aid of an automatic correction algorithm (Ansari and Kim 2007), the high-resolution scheme minimized the numerical discretization errors. The measurement for the convergence was the root mean square (RMS) residual value of 10^−7^.

The variance of the liquid species was calculated at a cross-section of the micromixer normal to the flow direction, which can be articulated mathematically (Xia et al. 2005) as follows:

4 $${{\upsigma }} = \sqrt {\frac{1}{\text{N}}\mathop \sum \limits_{i = 1}^{N} ({\text{c}}_{i} - {\bar{\text{c}}}_{\text{m}} )^{2} }$$

where *N* is the number of sampling points within the plane, *c*_*m*_ and *c*_*i*_ signify the optimal mass fraction and mass fraction at sampling point *i*, respectively. At any cross-sectional plane, optimal mass fraction (*c*_*m*_) is 0.5. To investigate the mixing performance of the micromixer quantitatively, the mixing index at a specific cross-sectional plane is defined using the variance (Kockmann et al. 2003) as:

5 $${\text{M}} = 1 - \sqrt {\frac{{{{\upsigma }}^{2} }}{{{{\upsigma }}_{ \hbox{max} }^{2} }}}$$

where $\sigma$ is the standard variance of the concentration at particular cross-sectional plane and $\sigma_{max}$ is the maximum standard variance all over the data range. The maximum and minimum values of the variance are obtained for entirely unmixed and entirely mixed fluids, respectively. The mixing index varied from 0 to 1 (for complete mixing). The higher mixing index represents a better homogeneous concentration and higher mixing performance. In this study, the Reynolds number (Re) and Péclet number (Pe) were defined as follows;

6 $${\text{Re}} = \frac{{{{\uprho V L}}}}{{{\upmu }}}$$

7 $${\text{Pe}} = \frac{\text{V L}}{\text{D}}$$

where L is the hydraulic diameter of the inlet channel, V is the inlet velocity, µ is the dynamic viscosity of the water, and D is the diffusivity.

## Results and discussion

**Fig. 2:**
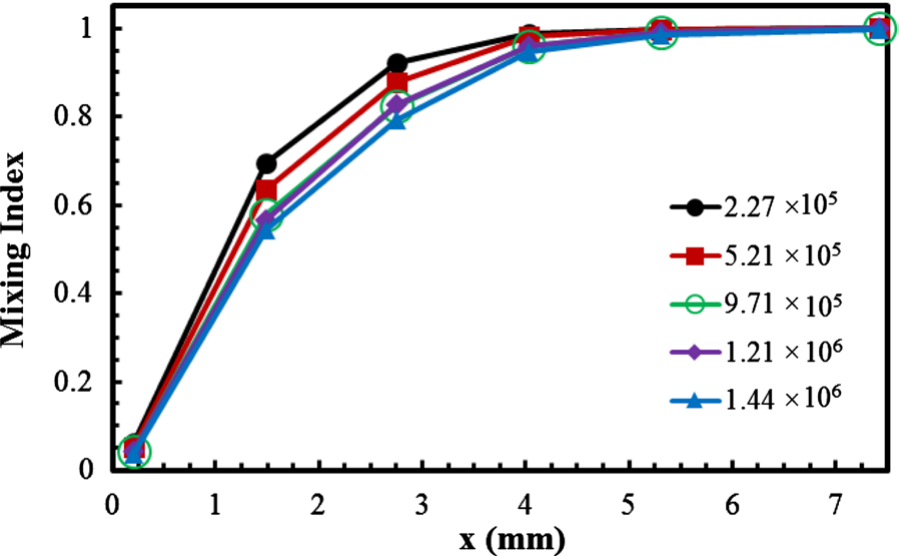
Grid dependency test for development of mixing index (Re = 40)

**Fig. 3:**
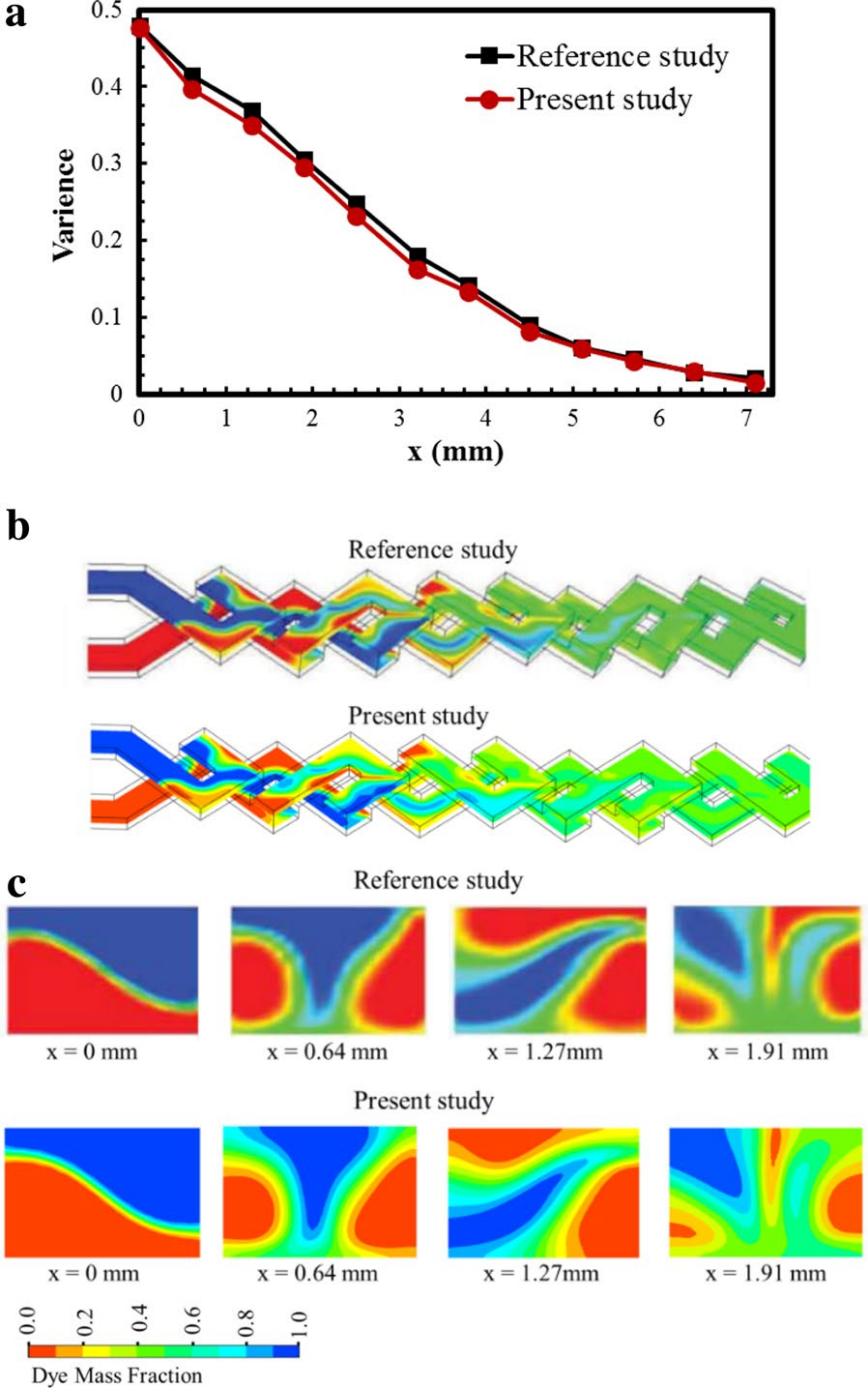
Comparison of present computational results with previous results (Xia et al. 2005) for the reference model at Re = 0.2: **a** distributions of the standard deviation along the channel, **b** dye mass fraction distributions on x–y planes at the middle of top and bottom channels, and **c** dye mass fraction distributions on the y–z planes

A preliminary investigation of grid sensitivity was carried with five numbers of nodes in a range from 2.26 × 10^5^ to 1.44 × 10^6^ as shown in Fig. 2. The test was performed at Re = 40 for the reference design. The mixing index was computed along the length of the micromixer. From the test results, the grid system with 1.21 × 10^6^ nodes was selected for further analysis. Present computational results were compared with the previous results (Xia et al. 2005) quantitatively and qualitatively as shown in Fig. 3. Figure 3a represents the distributions of the variance of the mass fraction along the channel length at Reynolds number 0.2. The variance of the mass fraction varies from 0 (for complete mixing) to 0.5 (for incomplete mixing). The variance decreases gradually along the channel in both cases, and the present numerical results show good agreements with the previous numerical results. In Fig. 3b, c, distributions of the dye mass fraction are presented. In the present study, the x–y planes (Fig. 3b) are taken at the middle depths of the top and bottom channels, whereas the previous work (Xia et al. 2005) did not described the exact locations of the planes. Distributions of the dye mass fraction on the y–z planes have been compared along the channel length as shown in Fig. 3c. Qualitatively, both the figures (Fig. 3b and c) represent good agreements between these two numerical results.

**Fig. 4:**
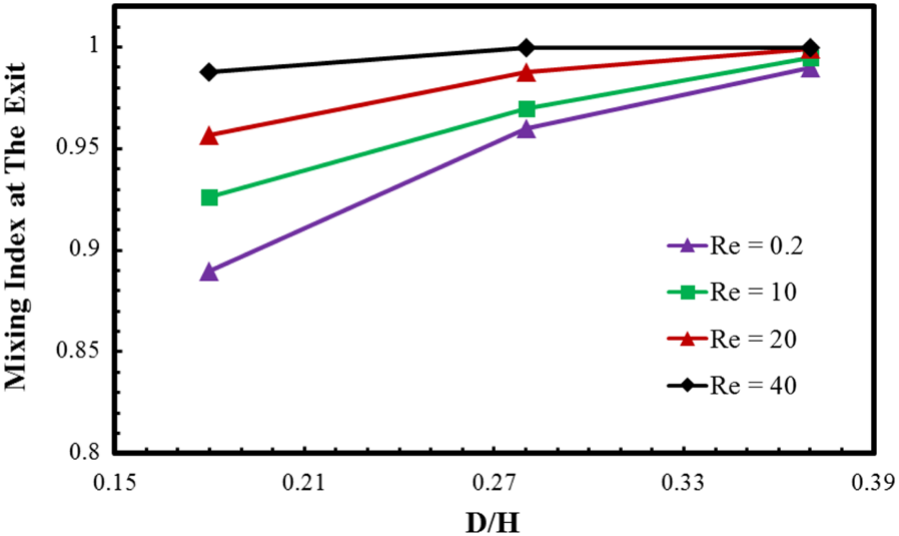
Effects of D/H and Re on the mixing index at the exit of the micromixer for W_C_/H = 0.28

Variations of the mixing index at the exit of the micromixer with D/H and Reynolds number are shown in Fig. 4. As the Reynolds number increases, chaotic advection becomes active in the micromixer. It is observed that at the nodes of the crossing structure the fluids are driven from the bottom layer to the top layer through the rotation and rejoin to its main flow, which involves stretching and folding of fluid layers, and consequently chaotic advection is generated promoting mixing of the fluids. The results shown in Fig. 4 also represent that, as the Reynolds number increases, the mixing index at the exit also increases. At Reynolds number 0.2, 10, and 20, the mixing index increases with the increase in D/H ratio, while at the highest Reynolds number (Re = 40) the effect of D/H on the mixing index is almost negligible. At the highest value of D/H ratio (D/H = 0.37), difference in the mixing index among the case with four Reynolds numbers becomes very small (Mo = 0.990, 0.995, 0.999, and 0.999 at Re = 0.2, 10, 20, and 40, respectively, where Mo is the mixing index at the exit). The mixing index becomes more sensitive to D/H as Reynolds number decreases. At Re = 0.2, with the increase in D/H from 0.18 to 0.37, the mixing index increases from 0.89 to 0.99, which is an excellent achievement in mixing at this low Reynolds number.

**Fig. 5:**
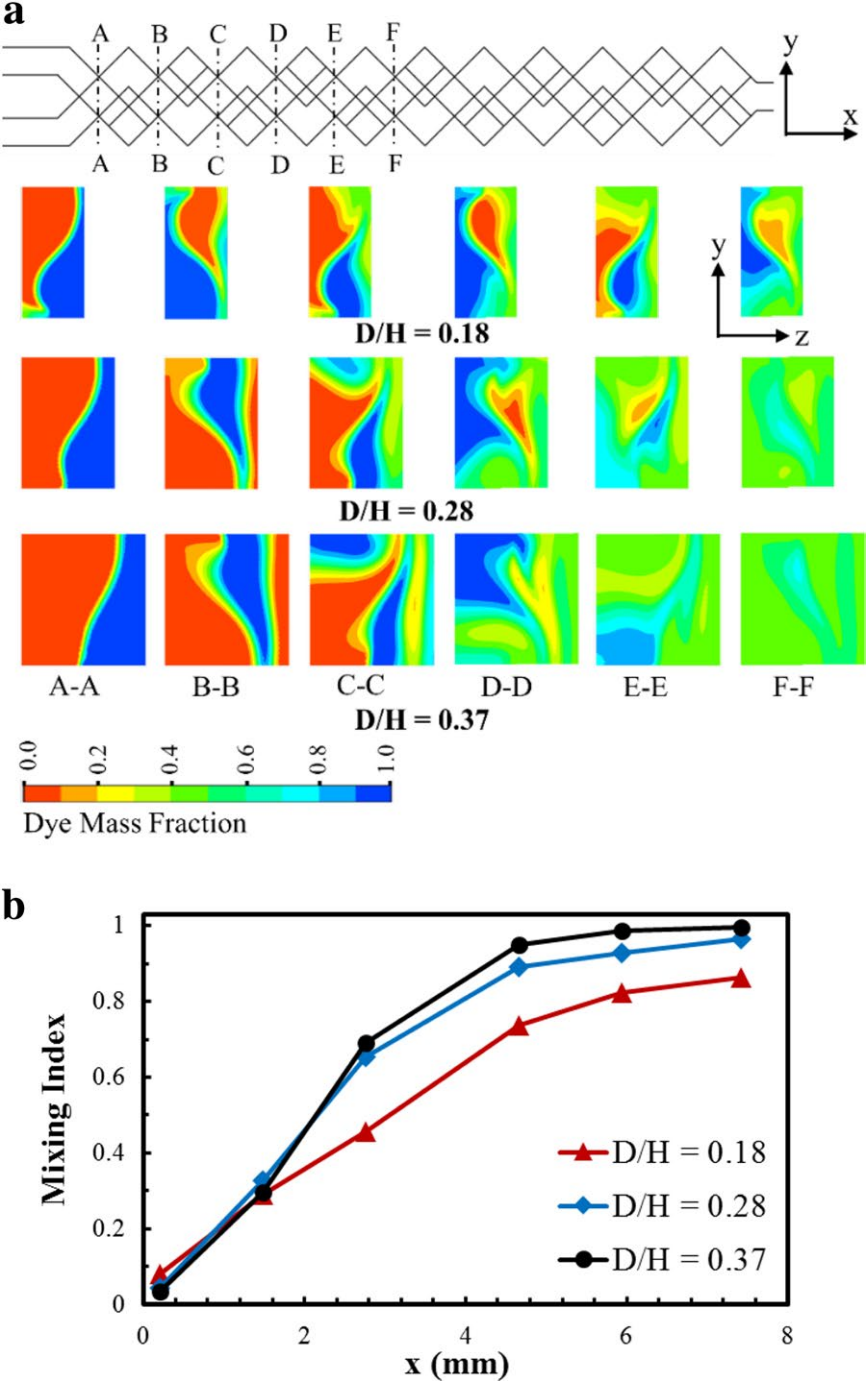
Mixing in proposed micromixer at various Reynolds numbers and D/H (W_C_/H = 0.28 and Re = 0.2): **a** Qualitative representation of dye mass fraction at six cross-sectional planes (A–A to F–F) for three different D/H, **b** Developments of the mixing index along the channel length for different D/H

Effects of D/H on chaotic mixing mechanism were analyzed both quantitatively and qualitatively in Fig. 5. The dye mass fraction distributions on consecutive cross-sectional planes along the axis of the micromixers (indicated by dotted lines), are shown in Fig. 5a for Reynolds number, 0.2. As the flow proceeds, the fluid layers are subdivided gradually into the thinner layers by the stretching and folding of fluids due to chaotic advection, and the interfacial area between the fluids extends greatly promoting mixing performance. With the change in the channels depth, Reynolds number was kept constant by adjusting the velocity at the inlets. Therefore, the residential time of fluids in the micromixer, which is inversely proportional to the inlet velocity, increases with D/H. Because of the highest residential time, the micromixer with D/H = 0.37 confirms the maximum mixing performance. Figure 5b represents the development of mixing performance in the micromixer at Re = 0.2 for different D/H values. This figure shows that the mixing index increases along the channel length, and beyond some inlet length the mixing index also increases with D/H.

A quantitative comparison of the present result for the mixing index with those reported by the previous studies on various shapes of micromixers is summarized in Table 2. This comparative review reveals that the present micromixer shows the extraordinarily high mixing index at Re = 0.2 compared to the previous micromixers (Ansari and Kim 2009; Hossain and Kim 2014, 2015; Alam and Kim 2012). The relatively long channel length seems a minor contribution to this mixing index.

**Table 2:** Comparison of present results on mixing index with those of previous studies

Sr. no	Investigators	Geometrical shapes	Design parameter	Hydraulic Dia. (mm)	Reynolds number	Mixing length (mm)	Mixing index (Mo) variations in the tested range
1	Ansari and Kim (2009)	3D serpentine	straight channel length to the channel width (s/d)	0.15	1.0	12	0.23–0.25
2	Hossain and Kim (2014)	Unbalanced three split and recombine	rhombic angle (θ)	0.15	0.1	4.0	0.23–0.31
3	Hossain and Kim (2015)	3D serpentine split and recombine	sub-channel width to the main channel width (w/W)	0.13	0.1	2.1	0.42–0.43
4	Alam and Kim (2012)	Curved microchannel with rectangular grooves	groove depth to the main channel width (d/W)	0.1	0.5	2.66	0.20–0.21
5	Present study	Two-layer crossing microchannels	channels depth to the main channel width (D/H)	0.2	0.2	7.2	0.89–0.99

**Fig. 6:**
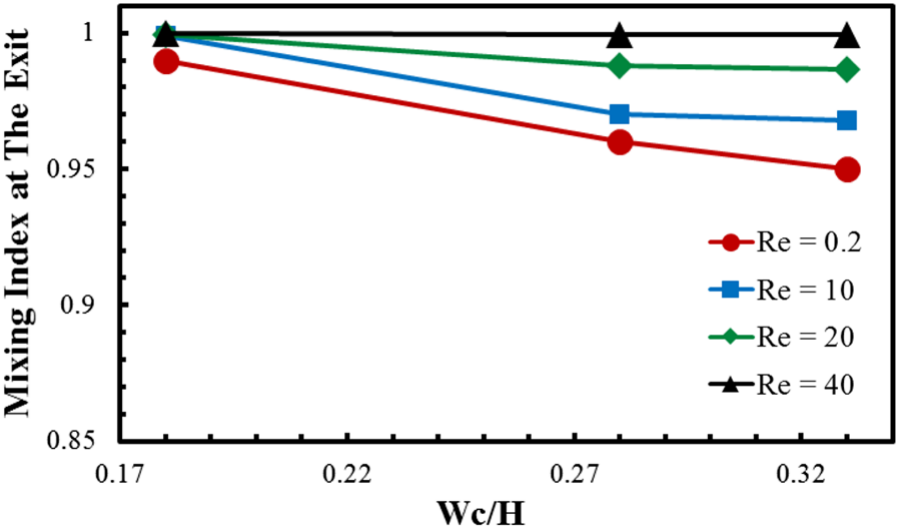
Effects of Wc/H and Re on the mixing index at the exit of the micromixer for D/H = 0.28

**Fig. 7:**
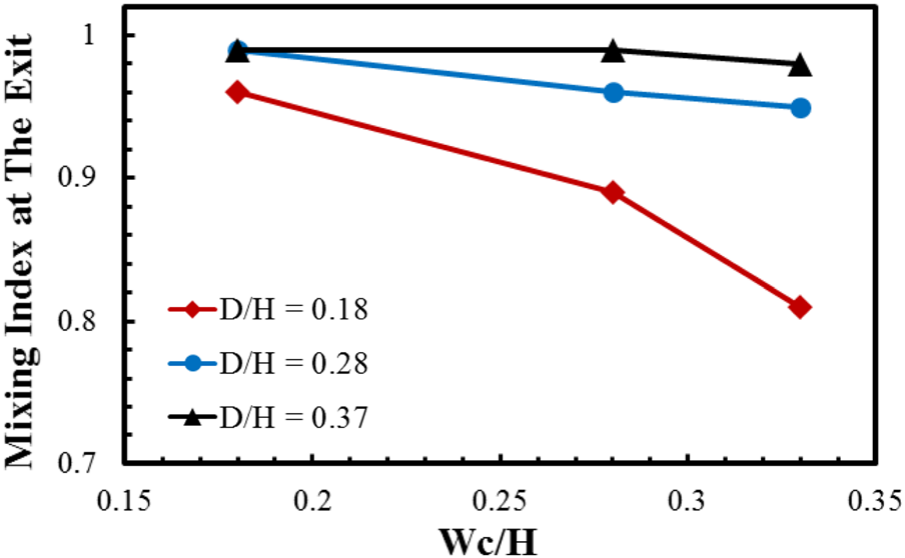
Effects of Wc/H and D/H on mixing performance at the exit of the micromixer at Re = 0.2

Mixing index variations at the exit of the micromixer with Wc/H and Reynolds number are shown in Fig. 6. Mixing index at the exit decreases with increase in Wc/H. However, at lowest value of Wc/H (= 0.18), the effect of Reynolds number on mixing index is almost negligible. At the highest Reynolds number (Re = 40), mixing index at the exit converges at 0.99 regardless of Wc/H. To analyze the coupled effects of Wc/H and D/H on the mixing performance, variations of the mixing index at the exit of the micromixer are plotted at Re = 0.2 in Fig. 7. Axial distance of the micromixer was maintained constant for the parametric study. With the increase in the sub-channel width (Wc), the lengths of Lc and Ls decrease as shown in Fig. 1, which reduces the total flow path and thus also reduces the residence time. Therefore, the mixing index decreases as Wc/H increases except at D/H = 0.37, where the variation of the mixing index is negligible. As a result, at D/H = 0.18, the mixing index reduces to 0.81 at Wc/H = 0.33.

**Fig. 8:**
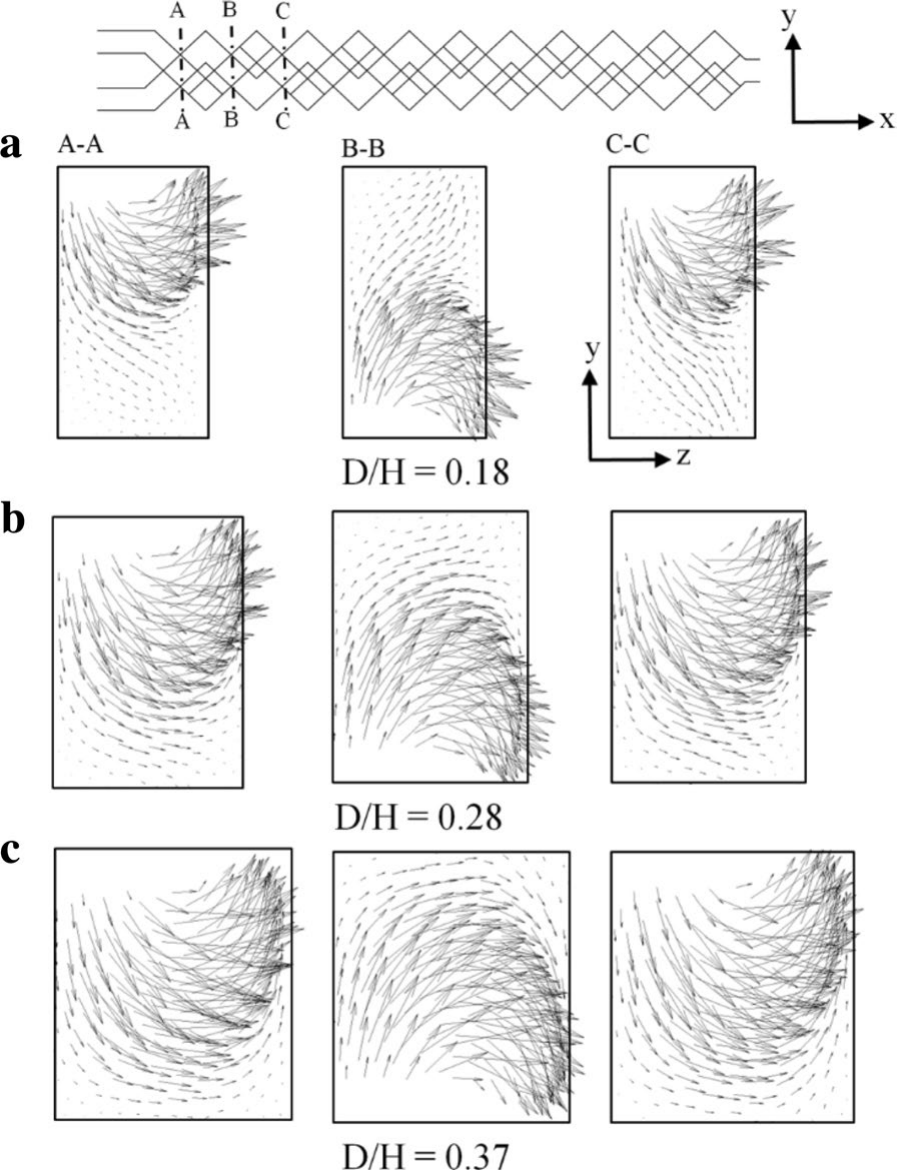
Velocity vector plots on y–z planes for Wc/H = 0.28 at Re = 0.2: **a** D/H = 0.18, **b** D/H = 0.28, and **c** D/H = 0.37

Figure 8 illustrates the velocity vectors on y–z planes (indicated by dotted lines) with three different D/H at Re = 0.2. The velocity vectors are plotted at the successive cross-sectional planes. As the flow proceeds from section A–A to C–C, the identical flow structures are visualized in the alternate planes for all three values of D/H. Due to the overlapping crisscross structure, the velocity vectors visualize the grouping of hyperbolic and helical flow structures over the cross-sectional area at D/H = 0.18 (Fig. 8a). However, with the increase in D/H, area of the helical motion reduces. And, at D/H = 0.37 (Fig. 8c), the velocity vectors are visualized with a strong hyperbolic flow structure all over the cross-sectional plane, consequently the micromixer depicts higher mixing performance.

**Fig. 9:**
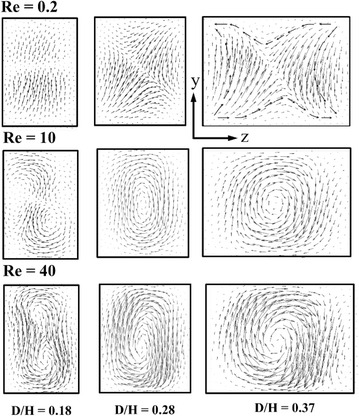
Velocity vector plots on y–z plane at the exit of micromixer for various Reynolds numbers and D/H at Wc/H = 0.28

Figure 9 shows that the velocity vector plots on the y–z planes at the exit of the micromixer for different D/H values at Re = 0.2, 10 and 40. An important feature that improves the mixing efficiency in the chaotic micromixer is ‘‘saddle-shaped’’ flow pattern (indicated by dotted lines), which plays a significant role in generating chaotic advection at the low Reynolds number (Re = 0.2). The “saddle-shaped” flow pattern can stretch the fluid stream about the hyperbolic center, and thus increase the interfacial area of the fluids (Xia et al., 2005). It is found that at Re = 0.2 the increase in D/H strengthens the ‘‘saddle-shaped’’ flow pattern. On the other hand, at Re = 10 and 40, Fig. 9 shows that circular or elliptical flow pattern in the cross-sectional planes instead of ‘‘saddle-shaped’’ flow pattern. At D/H = 0.37, a circular flow pattern is visualized at the center of the cross sectional plane, while an elliptical flow pattern is found at the other values of D/H. With the increase in Reynolds number from 10 to 40, the velocity vectors strengthen for fixed D/H, which increases the mixing index at the exit of the micromixer.

**Fig. 10:**
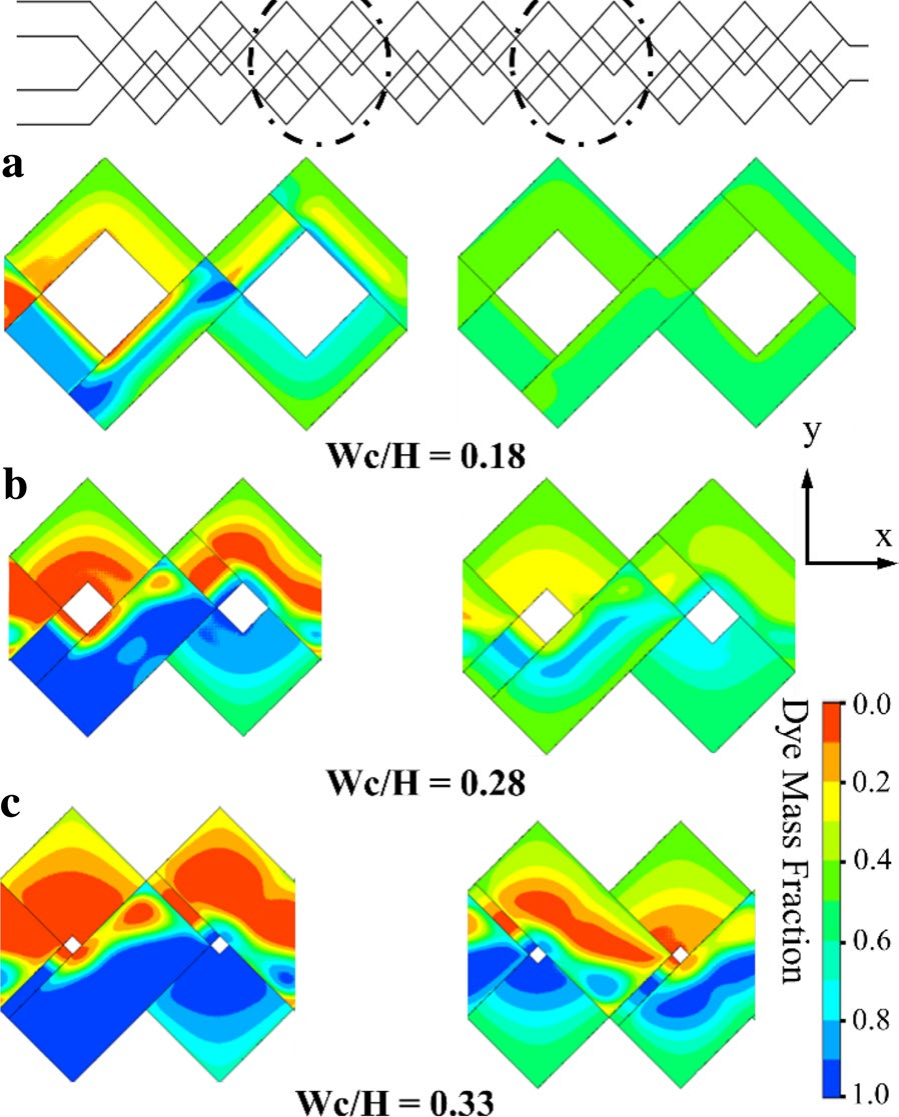
Dye mass fraction distributions on x–y planes for D/H = 0.28 at Re = 0.2: **a** Wc/H = 0.18, **b** Wc/H = 0.28, and **c** Wc/H = 0.33

**Fig. 11:**
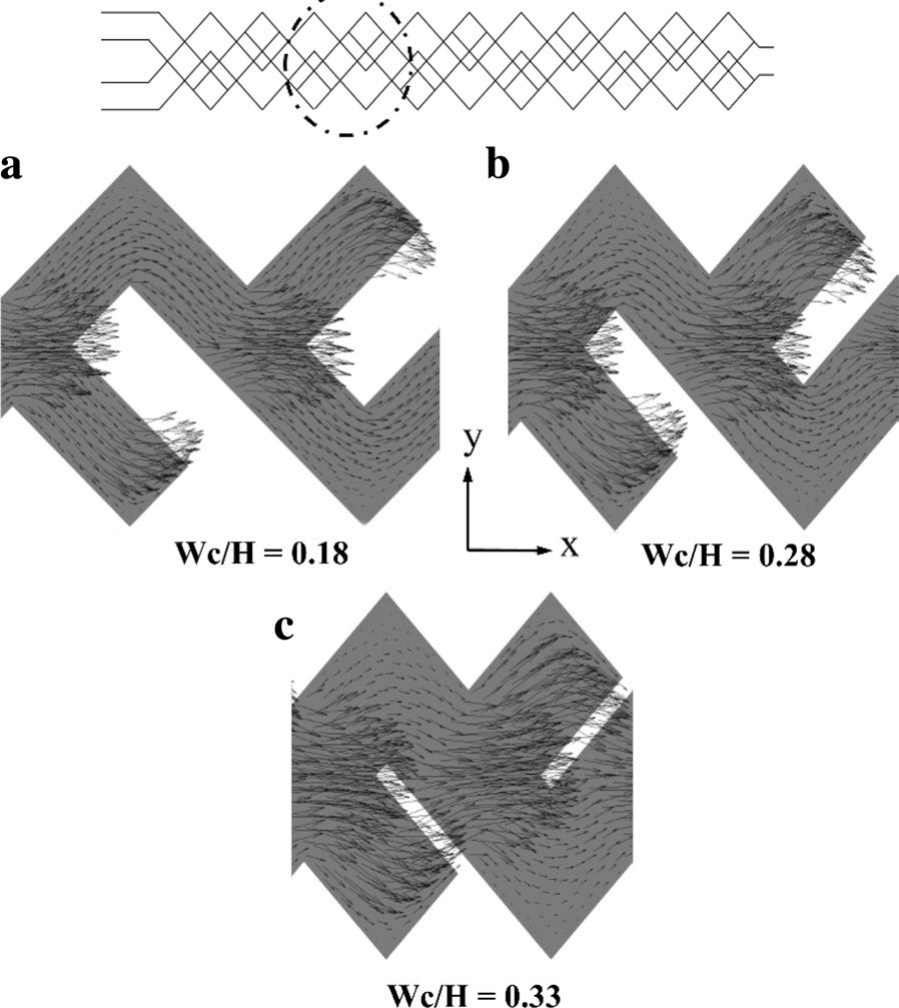
Velocity vector plots on x–y planes (indicated by *dotted circle*) at the middle of the channels (Re = 0.2)

For a qualitative comparison at different Wc/H ratios, the dye mass fraction distributions on x–y planes were plotted at Re = 0.2 in Fig. 10. Mass fraction distributions have been taken at the 2nd and 4th units (as indicated by the dotted circular lines) and at the middles of the top and bottom channels. As Wc/H increases, Lc and Ls shown in Fig. 1 decrease. Shorter lengths of Lc and Ls may reduce the chaotic advection in the micromixer. The micromixer with Wc/H = 0.18 shows almost complete mixing at 4th unit as depicted in Fig. 10a. To explain the effects of Lc and Ls on mixing, velocity vectors on x–y planes (indicated by dotted circle) at the middle of the top and bottom channels are plotted at Re = 0.2 in Fig. 11. Figure 11a at Wc/H = 0.18 shows that the velocity vectors are aligned along the channel in most part of the cross-sectional planes. However, for shorter lengths of Lc and Ls (larger W/H), the velocity vectors are not well aligned as shown in Fig. 11b, c. This phenomenon also affects the mixing index at the exit of the micromixer shown in Fig. 10.

**Fig. 12:**
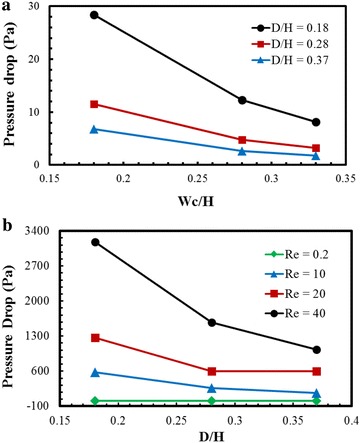
Effects of design parameters and Reynolds number on the pressure drop: **a** Wc/H at Re = 0.2 and **b** D/H at Wc/H = 0.28

The pressure drop characteristics were estimated in terms of design parameters and Reynolds number as shown in Fig. 12. The pressure drop in a microfluidic system is associated with the pumping power of the working fluids. The pressure drop was computed as the difference in the area-weighted average pressure between the inlet and exit of the micromixer. Figure 12a presents the effect of Wc/H values on the pressure-drop at Re = 0.2 for the three values of D/H, i.e., 0.18, 0.28, and 0.37. The pressure-drop decreases as Wc/H increases for all values of D/H. And, at fixed values of Wc/H, the pressure drop increases as D/H decreases. Thus, the pressure drop reaches the maximum at the lowest values of D/H and Wc/H. Figure 12b shows the effects of Reynolds number and D/H on the pressure drop at Wc/H = 0.28. The pressure drop decreases with increase in D/H except at Re = 0.2, where the pressure drops remain invariant. This figure confirms that the micromixer with lower channels depth shows higher pressure drop.

**Fig. 13:**
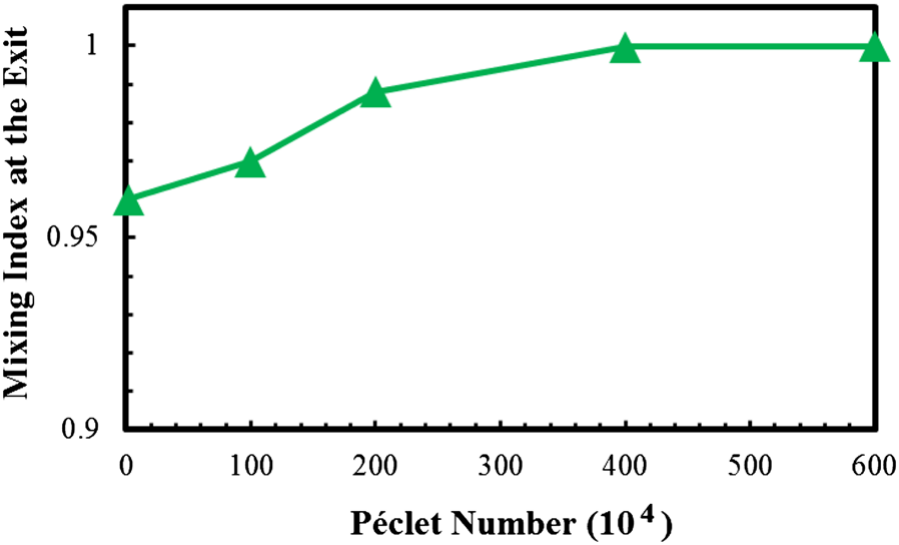
Variation of mixing index at the exit of micromixer with Péclet number

The diffusion effect on mixing index can be represented by the Péclet number (Pe) defined by Eq. (7). Figure 13 shows the variation of mixing index at the exit of the reference micromixer in a range of Pe less than 6.0 × 10^6^. The mixing index increases as Pe increases up to 4.0 × 10^6^, but further increase in Pe does not change the mixing index. This indicates that the effect of molecular diffusion on the mixing of fluids in this micromixer is negligible for Péclet numbers larger than 4.0 × 10^6^.

## Conclusions

This work presents a parametric investigation on flow structure and mixing in a micromixer with two-layer crossing channels which was reported by Xia et al. (2005). The flow and mixing performance were numerically analyzed using ANSYS-CFX inbuilt diffusion–convection model. Present numerical results for mixing agree quantitatively and qualitatively well with the previous numerical results. The mixing index along with pressure-drop have been examined in terms of two geometric parameters related to sub-channel width and depth, i.e., Wc/H and D/H, respectively, at various Reynolds numbers in a range from 0.2 to 40. The mixing index at the exit of the micromixer increases with the increase in D/H, and becomes more sensitive to D/H as Reynolds number decreases. At Re = 40, the effect of D/H on the mixing index is almost negligible, while at Re = 0.2 with the increase in D/H from 0.18 to 0.37, the mixing index at the exit of the micromixer increases from 0.89 to 0.99, which is quite good achievement in mixing at this low Reynolds number. A quantitative comparison among the mixing indexes at low Reynolds numbers in various micromixers indicates that the present micromixer produces the extraordinarily high mixing index at Re = 0.2 compared to the previous micromixers. Velocity vectors at Re = 0.2 show that increase in D/H strengthens the ‘‘saddle-shaped’’ flow pattern. The mixing index at the exit of the micromixer generally decreases with increase in Wc/H, and the decreasing rate of the mixing index with Wc/H becomes larger as D/H and Reynolds number decrease. At Re = 0.2, the maximum relative reductions in pressure drop are about 70 and 75 %, respectively, with the increase in Wc/H and D/H in their tested ranges. This study is expected to provide preliminary input for the design of an optimum micromixer.
